# Evaluation of options for harvest of a recombinant *E. Coli* fermentation producing a domain antibody using ultra scale‐down techniques and pilot‐scale verification

**DOI:** 10.1002/btpr.2220

**Published:** 2016-01-12

**Authors:** Ioannis Voulgaris, Alex Chatel, Mike Hoare, Gary Finka, Mark Uden

**Affiliations:** ^1^ Dept. of Biochemical EngineeringThe Advanced Centre for Biochemical Engineering, UCLGower StLondonWC1E 6BT; ^2^ Biopharm Process Research, BioPharm R&D, GlaxoSmithKline, R&DStevenageSG1 2NY

**Keywords:** fermentation, centrifugation, filtration, flocculation, domain antibody, E. coli

## Abstract

Ultra scale‐down (USD) methods operating at the millilitre scale were used to characterise full‐scale processing of *E. coli* fermentation broths autolysed to different extents for release of a domain antibody. The focus was on the primary clarification stages involving continuous centrifugation followed by depth filtration. The performance of this sequence was predicted by USD studies to decrease significantly with increased extents of cell lysis. The use of polyethyleneimine reagent was studied to treat the lysed cell broth by precipitation of soluble contaminants such as DNA and flocculation of cell debris material. The USD studies were used to predict the impact of this treatment on the performance and here it was found that the fermentation could be run to maximum productivity using an acceptable clarification process (e.g., a centrifugation stage operating at 0.11 L/m^2^ equivalent gravity settling area per hour followed by a resultant required depth filter area of 0.07 m^2^/L supernatant). A range of USD predictions was verified at the pilot scale for centrifugation followed by depth filtration. © 2016 The Authors Biotechnology Progress published by Wiley Periodicals, Inc. on behalf of American Institute of Chemical Engineers *Biotechnol. Prog.*, 32:382–392, 2016

## Introduction

*E. coli* is widely used for the production of proteins and especially antibody fragments that do not require glycosylation.^1^ Because *E. coli* lacks the ability to secrete recombinant protein into the extracellular space, cell lysis is required for release of the recombinant product. However cell lysis can have a detrimental effect on the downstream processing, for example, increased viscosity due to release of cytoplasmic DNA,^2^ reduced product titre due to cytoplasmic protease release,^3^ increased purification burden due to additional release of endotoxins.^4^ Approaches used to overcome these challenges include selective release from the periplasmic space,^2^, ^5^, ^6^ use of *E. coli* constructs with reduced protease activity,^7^ use of added or constitutively expressed nucleases for DNA degradation,^8^ selective release to the extracellular space using secretion tags.^9^, ^10^ However, these are all faced with processing challenges, for example, the need to avoid premature cell lysis during fermentation,^9^ the changes in cell physiology due to changes in protein expression^9^, ^11^ and the need to remove selectively secretion tags.^12^ Alternative approaches include the use of mechanical homogenisation, which leads to micronization of cell wall debris and hence difficult separation,^13^, ^14^ or the use of selective agents for precipitation of the DNA and flocculation of the cell debris and endotoxins.^15^, ^16^ The advantage here is the relatively rapid action of reagents such as polyethyleneimine (PEI) to achieve selective precipitation and flocculation and the rapid separation possible by use of high speed centrifugation. Hence rapid processing of the lysed broth should be possible leading to a relatively stable clarified broth, which can be quickly processed or stabilized to reduce action of residual proteases.

Flocculation of cells at harvest is often used to aid clarification; this is usually achieved with polyionic polymers (such as PEI, diethylethanolamine, dextran, and acryl‐based polymers), polydiallyldimethylammoniun chloride,^17^ or with inorganic materials such as diatomaceous earth and perlite,^18^ or chitosan (sourced from crustaceans) or cationic polysaccharides of starch and inulin.^19^ PEI has been shown to have a range of effects on a lysed microbial cell suspension including the precipitation of colloidal proteins, endotoxins, and nucleic acids and the flocculation of cell debris material.^20^ A key impact is the resultant clarification by centrifugation with not only the removal of the cell debris material but also the removal of the additional solids created by precipitation. A recent study of PEI treatment of *E. coli* lysates showed that PEI was effective in removal by precipitation of nucleic acids and colloidal proteins and by flocculation of submicron particles such as cell debris and intracellular components from lysed cells.^21^ This was even after the flocculated/precipitated suspension had been subjected to extreme conditions of shear stress, an important result when considering the translation from bench scale to pilot or manufacturing scale operations.^22^ The challenges involved in the use of reagents such as PEI include the need to adjust the level of addition according to the concentration of impurities present, the shear sensitive nature of the flocs and precipitates formed and the need to demonstrate removal of the reagent to acceptable levels in the subsequent purification stages.^18^

One option for the clarification of lysed microbial cell broths is continuous flow high speed centrifugation^22^, ^23^ as a preparative stage for depth filtration. Key factors affecting the performance include the imparted shear stress in the centrifuge feed zone and the imposed “centrifugal force” and the residence time available for separation. A well–established Sigma theory is available to classify various centrifuge types and scales in terms of equivalent separation area available with the demonstration of approximately equivalent clarification for machines operating under similar ratios of flow rate to centrifugation area.^24^ Ultra scale‐down (USD) shear devices have been created to mimic the exposure to shear stress in the centrifuge feed zone, for example, rotating disc configurations^25^, ^26^ or capillary flow configurations.^27^ These have been followed by bench‐top, test‐tube centrifugation to evaluate the ease of recovery by centrifugation. This combination of bench scale devices has been successfully used to predict the performance of industrial‐scale machines.^23^, ^25^, ^28^, ^29^

For the subsequent depth filtration stage, the challenge is to understand how the process material (centrifuge supernatant) interacts with the filter media and the impact on the flux rate versus pressure relationship. Recent studies have employed constant pressure devices operated on automated platforms to determine the flux versus time characteristic in terms of a resistance model, for example, for the membrane filtration of *E. coli* broths^30^ or a pore constriction model, for example, for the depth filtration of mammalian cell broths.^31^ These are just two of many approaches to characterization of a filtration process.^32^ The translation from estimates of filter capacity from constant pressure operation to constant flux operation requires careful consideration of operating conditions being used. The USD studies help provide insight of how the process material interacts with filters to help enable prediction of the full scale operation.

A domain antibody expressed in *E. coli* provides the system of study in this article. Domain antibodies have potential both as diagnostic and therapeutic agents, for example, their small size allows easier penetration in poorly vascularised tissues and they provide a basis as building blocks, for example, for antibody‐drug conjugates or diabodies. A challenge is posed by their purification from one of the key hosts for manufacture, that is, *E. coli*, with issues such as clarification of lysed broths posing major demands on downstream processing operations such as centrifugation and filtration due to the relatively small size of particulate material to be removed and the wide range of interfering contaminants to be processed. A recent study^2^ has demonstrated the role of flocculation in facilitating the reduction in levels of debris, colloidal protein, lipopolysaccharides, and nucleic acids as an early clean up stage prior to high resolution chromatography.

In this study, USD tools and methodologies are used to study the interface between fermentation, harvesting, and the early stages of downstream processing i.e. primary clarification prior to chromatography. In particular, the role of combined precipitation and flocculation is explored to enable this sequence.

## Materials and Methods

### Cultivation and bioreactor settings

The *E. coli* W3110 (ATCC^®^ 27325^TM^) strain is used with plasmid pAVE011 harbouring a TNFR1 dAb, a V_H_ domain antibody of approximately molecular weight 13.1 kDa with OmpA leader sequence for secretion to the periplasm. The cells were stored in glycerol (20%, v/v) at −80°C. 1 mL lots of glycerol stock were inoculated in 1000 mL baffled flasks (UltraYield FlaskTM, Thomson Instrument Company, Kent, UK) containing 400 mL of vLB inoculation medium. The flasks were grown in a rotary‐shaking incubator at 220 rpm and 37°C until OD_600_= 1.0. This method of inoculum preparation was used for all bioreactors to avoid seed culture variability; inoculation was carried out at 1:50 volume ratio for both scales of fermentation studied.

**Figure 1:**
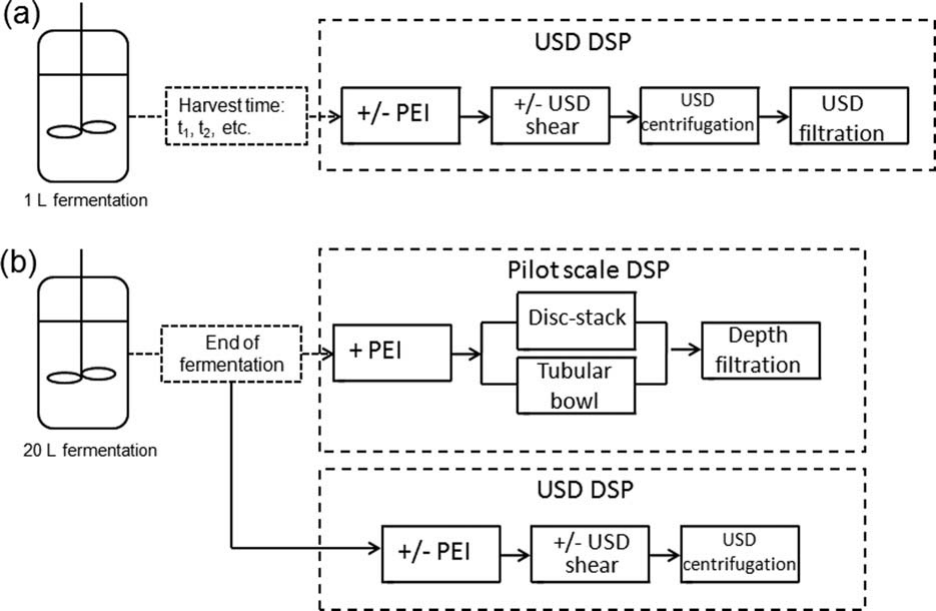
**Process studies undertaken.** Flowsheets show (a) the USD study of the broth processability as a function of harvest time; (b) pilot scale study of recoverability of flocculated broth with complementary USD study of centrifugation stage.

The bench‐scale fermentations were carried out using a parallel bioreactor system of 1 L working volume reactors (SR1000DLL bioreactor, vessel diameter 100 mm, aspect ratio 2.4:1, overhead driven triple Rushton 6‐blade 46 mm dia impellers (DASGIP AG, Jülich Germany) with data recorded using DASGIP Control. DO control was by a cascade system, which increased sequentially impeller speed (400–1600 rpm), then gas flow rate (1–2 vvm) and then oxygen content in the gas (21–100%). The capacitance of the culture was measured online at 1000 KHz (Aber Instruments, Aberystwyth, UK); 80 mL samples of the fermentation broth were collected at predetermined time points postinduction. The samples were processed for USD studies as described in Figure 1.

The pilot‐scale fermentations were carried out using a 20 L working volume bioreactor (vessel diameter 240 mm, aspect ratio 3:1, overhead driven triple 6‐blade Rushton impeller (95 mm dia, 20 < *N* < 1000 rpm), Sartorius BIOSTATR C plus, Sartorius AG, Goettingen, Germany). The DO set‐point was maintained at 30 ± 5% by a cascade system, which increased sequentially impeller speed (300–900 rpm), then gas flow rate (1–2 vvm) and then oxygen content in the gas (21–100%). The *E. coli* cells were cultured in a semicomplex medium containing glycerol as the main carbon source and yeast extract. The pH was kept at 7.0 ± 0.05 by automatic addition of 25% (v/v) NH_4_OH and 2 M H_3_PO_4_. The temperature was maintained at 30°C throughout the process. The DO set‐point was set at 30 ± 5%. The DO spike, observed on completion of glycerol consumption, was used to initiate a concentrated glycerol feed (714 g/L) containing yeast extract at a constant rate of 3.6 mL/L/h (giving a final broth volume increase of 10%; the broth analyses presented here are per unit volume of actual broth to be processed, that is, they are not corrected back to the original volume). When the cell broth reached OD_600_ = 80 ± 2, IPTG was added to a final concentration of 150 μM in the broth to induce the formation of dAb. Fermentation was completed at 50 h postinduction, that is, when significant degree of autolysis had taken place. Real time values of pH, dissolved oxygen, agitation speed, temperature, air‐flow rate, oxygen percentage, oxygen uptake rate, carbon dioxide evolution rate during fermentations were recorded automatically by the bioreactor software (MFCS, Sartorius AG, Göttingen, Germany).

### Floc preparation

In this article, a fixed dose of PEI is employed rather than a titration performed to determine the optimum dose (as described previously^22^); this is to allow rapid processing to be achieved. In all cases, the broth used had a wet biomass concentration ranging from 110 to 172 g/wcw/L.

PEI *(C_2_H_5_N)_n_*, *M*_W_ = 50,000–100,000, (Sigma‐Aldrich) water based solutions of concentration ranging from 2.5% (w/v) to 50% (w/v) for screening studies and 12.5% (w/v) for rest of studies were prepared with gentle mixing with DI water as diluent for 1 h at 23°C. For screening studies, the test material was fully autolysed broth as obtained at final harvest. PEI concentration ranged from 0.1 to 6.0% (w/v of broth) for the screen. A final PEI concentration of 0.5% (w/v of broth) was used for the remainder of the studies. All PEI concentrations were also recorded as g/g wcw.

For the screening studies, the PEI solution, 2.0 or 6.0 mL, was gently pipetted into the cell broth over 20 s at the tip of the impeller in a 100 mL baffled reactor fitted with an impeller (6 blade Rushton turbine, dia, *d_i_*, 20 mm, *N* of 1150 rpm, *Re* = 2000, $G={\left(P/V\mu \right)}^{0.5}=800\;$ s^−1^ where *G* is mean velocity gradient, *P/V* is power dissipated per unit volume and μ is suspension viscosity, *P = Po ρ N^3^ d_i_* 5 where *Po* = 5.7) to reach the required final PEI concentration. The solution was left to mix for *t* = 0.5 h at 21°C before processing ( Gt∼3×10>6>105 required for full floc strength to be gained^33^). Cell broth was flocculated immediately after harvest. The flocculated material was used for bioprocessing studies within 0.75 h.

At pilot scale, flocculation was carried out in the bioreactor operating at 300 rpm; 0.8 L of a 12.5% (w/v) PEI solution was added to cell broth at 40 mL/min via the feed port to a final PEI concentration of 0.5% (w/v of broth). The solution was left to mix for 1 h at 23°C and then diluted to give a suspension containing 7% packed (v/v; defined by centrifugation at 3100 max RCF for 5 min) prior to USD or pilot scale centrifugation studies. The flocculated broths were held for 2 h before further experimentation. The floc preparation and hold times were chosen to allow practical processing at scale.

### USD centrifugation

Samples were exposed to shear stress for 20 s in a rotary disc device (20 mL stainless steel chamber of 50 mm internal diameter and 10 mm height, fitted with a stainless steel rotating disc of 40 mm diameter and 0.1 mm thickness with disc speed 0‐416 rps) controlled by a custom designed power pack (UCL Mechanical Workshop). The disc speed was related to maximum energy dissipation rates, *ε* (W/kg), using a computation fluid dynamic derived empirical correlation (ε = 1.68 × 10^−3^ *N* ^3.71^ where *N* is disc speed, revs s^−1^, 33 < *N* < 416; Zhang et al., to be published). The broth was exposed to shear stress equivalent to 0.045 × 10^6^ W/kg (*N* = 100 rps), 0.53 × 10^6^ W/kg (*N*= 200 rps), 2.59 × 10^6^ W/kg (*N* = 300 rps). Previous published work^25^, ^28^ used CFD to gain an understanding of the energy dissipation rates, which exist in disc stack and tubular bowl centrifuges. The range of energy dissipation rates applied at USD scale for a period of 20 s has been used to mimic the feed zones of various pilot‐scale centrifuges such as a hydro‐hermetic (low shear stress) and a nonhermetic (high shear stress) disc stack centrifuge, as well as a tubular bowl centrifuge (only the high shear stress version of the centrifuge was studied here). The sedimentation properties of sheared and nonsheared samples were characterized using a test tube centrifuge in terms of equivalent settling area (*Σ*_T_) using a method previously described^22^, ^31^, ^34^

(1) $$\Sigma_{T}=(V_{\mathrm{lab}}\omega^{2})/(2g\ln(2R_{o}/(R_{o}+R_{i})))$$

where *V*_lab_ is the volume of process material in the centrifuge tube, *ω* is the radial speed, *g* is acceleration due to gravity, *R_i_* and *R_o_* are the radii to the top and bottom of the settling suspension. Fixed volumes, *V*_lab_ = 2.0 mL, were centrifuged (Centrifuge 5415R, Eppendorf, Germany) at set speeds and for set times as given in the results. The supernatant was recovered taking care not to disturb the sediment; the OD_600_ was recorded and the supernatant stored frozen (−80°C) until needed for USD depth‐filtration experiments. Well‐clarified supernatant, that is, baseline value for clarification, was prepared by centrifugation for 0.5 h at maximum RCF of 16,110 (Centrifuge 5415R, Eppendorf, Germany, 13,200 rpm). All operations were carried out at 21°C. The centrifugation conditions were recorded in terms of values of *V*_lab_ */(tΣ*_T_ *)*. The solids remaining (*S*) was characterized by:

(2) $$S=(OD_{S}{}^{\_}OD_{O}\mathrm{)/(O}D_{F}{}^{\_}OD_{O})\times 100$$

where $OD_{S}$ is the optical density of the supernatant of the centrifuged sample under test, $OD_{O}$ is the optical density of the well‐clarified supernatant and is the optical density of the sample prior to centrifugation.

### USD filtration

This was performed on a liquid handling robotic platform (Tecan Freedom EVO_1_, fitted with a TeVacS two‐position vacuum filtration manifold, Tecan, Reading, UK). Custom made filter housings (Mechanical Workshop, UCL) were fitted with 2.8 × 10^−5^ m^2^ elements of depth filter media (Seitz^®^ K–Series Depth Filter Sheets (pore size range 0.05–0.2 μm), Pall Corporation, Portsmouth, UK). Each pre‐cut filter was wetted with 1.4 mL (equivalent to 50 L/m^2^) of DI water using the same conditions as for the experiment followed by 2.0 mL of process material. Vacuum filtration was carried out at Δ*P* = 100 mbar with the feed volume monitored at 5 s intervals.

### Pilot scale centrifugation

Two different centrifuges were used at pilot scale (see Figure 1b): (1) a disc stack centrifuge with a bowl containing 43 conical discs, gap width 0.5 mm, total volume 0.6 L, solids holding capacity 0.25 L, *Σ*_(__*c*__=0.4)_ *=* 680 m^2^, operated at 50 L/h to give (*Q/Σ*)_ds_ = 2.01 × 10^−8^ m/s (CSA‐1, GEA Westfalia Separator Group GMBH, Oelde, Germany) and (2) a tubular bowl centrifuge total volume 1.1 L, solids holding capacity 1 L, *Σ*_(__*c*__= 0.9)_ *=* 930 m^2^ operated at 60 L/h to give *(Q/Σ*)_tb_ = 1.78 × 10^−8^ m/s (PneumaticScaleAngelus CARR Powerfuge^TM,^ Barry‐Wehmiller family company, Cuyahoga Falls, OH). In each case, 10 L of feed was used. Steady state was assumed to have been reached within 5 bowl volumes and the time to solids breakthrough by when the solids holding capacity is predicted to have been reached. Supernatant for subsequent filtration trials was collected between these two times. Both centrifuge bowls were maintained at 23°C during operation.

### Pilot scale filtration

Pall Supracap^TM^ cartridge (0.1 m^2^, EKS SEITZ^®^ depth filtration medium, pore size range 0.05–0.2 μm, Pall Corporation, Portsmouth, UK) was used for pilot scale studies. A pressure gauge (Wika 316SS, Wika Instruments, Redhill, UK) was placed at the inlet of the filtration cartridge. Flow through the cartridge was dispensed by a pre‐calibrated 6 roller peristaltic pump (Watson‐Marlow 505 Du, Watson Marlow, Falmouth, UK). The cartridge was rinsed and wetted with 5 L (equivalent of 50 L/m^2^) DI water (Milli‐Q water purification system, Merck Millipore, Abingdon, UK) at 100 L/m^2^/h. The pump was set to provide a feed of 100 L/m^2^/h. The pressure was recorded until either (a) it reached 1.6 bar abs or (b) 10 L (≡ 100 L/m^2^) of feed material had passed through the filter.

### Analytical methods

#### dAb concentration

Protein A chromatography (HPLC Agilent 1200, Agilent Technologies UK, West Lothian, UK, fitted with a 1 mL HiTrap MabSelect^®^ Xtra, GE Healthcare Life Sciences, Buckinghamshire, UK) was used with 0.1 M pH 7.3 PBS buffer for loading and equilibration. Sample preparation involved centrifugation of 1.0 mL broth (24,200 RCF for 10 min) for extracellular dAb. For intracellular dAb the pellet was resuspended to 1.0 mL final volume with 50 mM Tris pH 8, subjected to 4 freeze‐thaw cycles (freezing in dry ice followed by incubation in a dry bath for 5 min at 37°C) and 2 freeze‐sonication cycles (freezing in dry ice followed by sonication for 15 min) using a sonication bath (Camsonix C275, Elma Electronic GmbH, Singen, Germany). The sonicated samples were centrifuged at 24,200 RCF for 10 min and the cell lysate recovered and tested for titre as described above. Samples were diluted in a defined fashion in equilibration buffer to a concentration of ∼0.1 mg/mL, filtered using a 0.22 µm PVDF syringe filter and then placed on a cooled auto‐sampler (4°C) for the duration of the analysis cycle. Elution was performed using a 13 mM HCl buffer at pH 1.9 with product eluted recorded at 280 nm. Calibration was performed using standard solutions of pure dAb (GSK, Stevenage, UK).

### Biomass

The biomass was measured at OD_600_ and gravimetrically as wet cell weight (wcw) and dry cell weight (dcw). 1.0 mL samples were centrifuged (24,200 RCF for 10 min), and the recovered pellets were resuspended and washed twice in distilled water to record wcw and then dried to constant weight (105°C for 24 h) to record dcw.

### DNA concentration determination

The concentration of the double stranded DNA in the extracellular environment was determined using a Qubit 2.0 Fluorometer (Invitrogen, Carlsbad, CA) according to manufacturer's instructions.

### Rheology

A cup‐and‐bob rheometer was used (Brookfield DV‐2+ viscometer fitted with spindle CP40, Brookfield Engineering Laboratories, MA), exposing 0.5 mL of treated cell broth samples to shear rates of 37.5–1500 s^−1^ in 7 increments with 30 s hold at each increment for increasing and decreasing shear sweeps.

## Results

**Figure 2:**
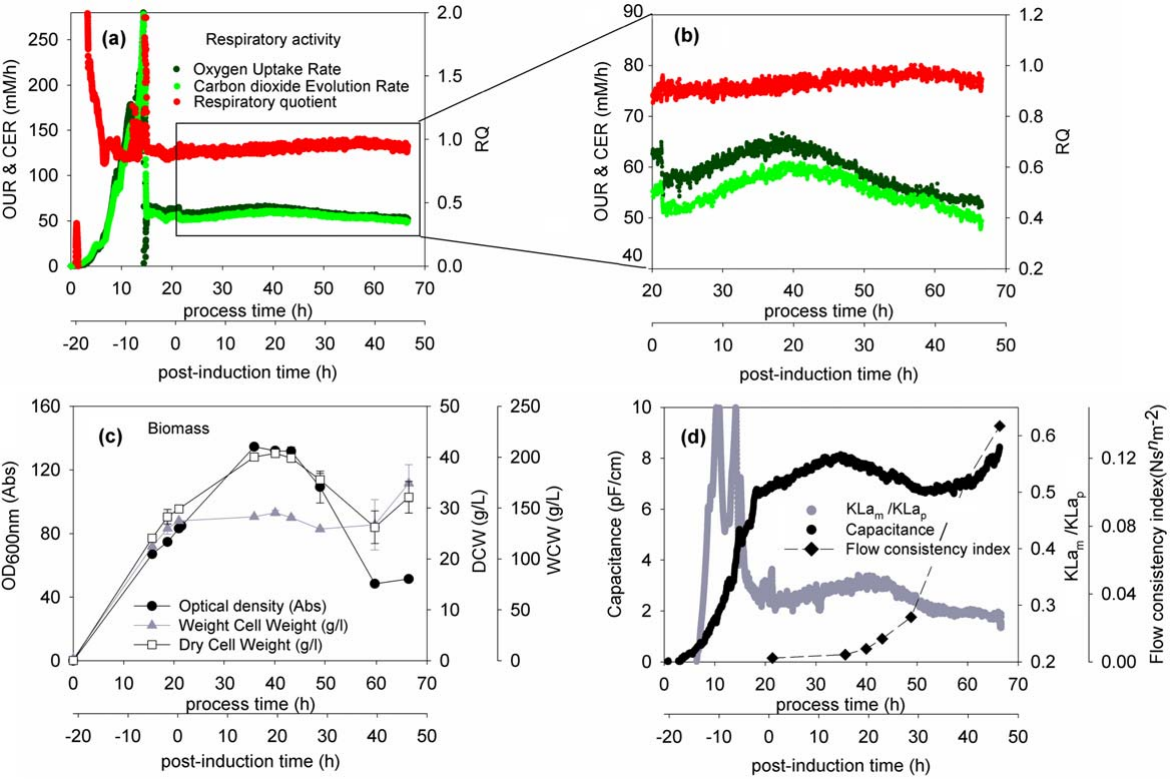
**Representative fermentation monitoring profiles for 1 L studies**. Given are (a) and (b) oxygen uptake rate, carbon dioxide evolution rate, and respiratory quotient; (c) biomass expressed as optical density at 600 nm, wcw, dcw; (d) culture capacitance at 1000 KHz, ratio of measured to predicted *K*_L_ *a* (see Results text) and viscosity expressed as flow consistency index (*n* ranges from 0.66 to 1.0 see Table 1).

**Figure 3:**
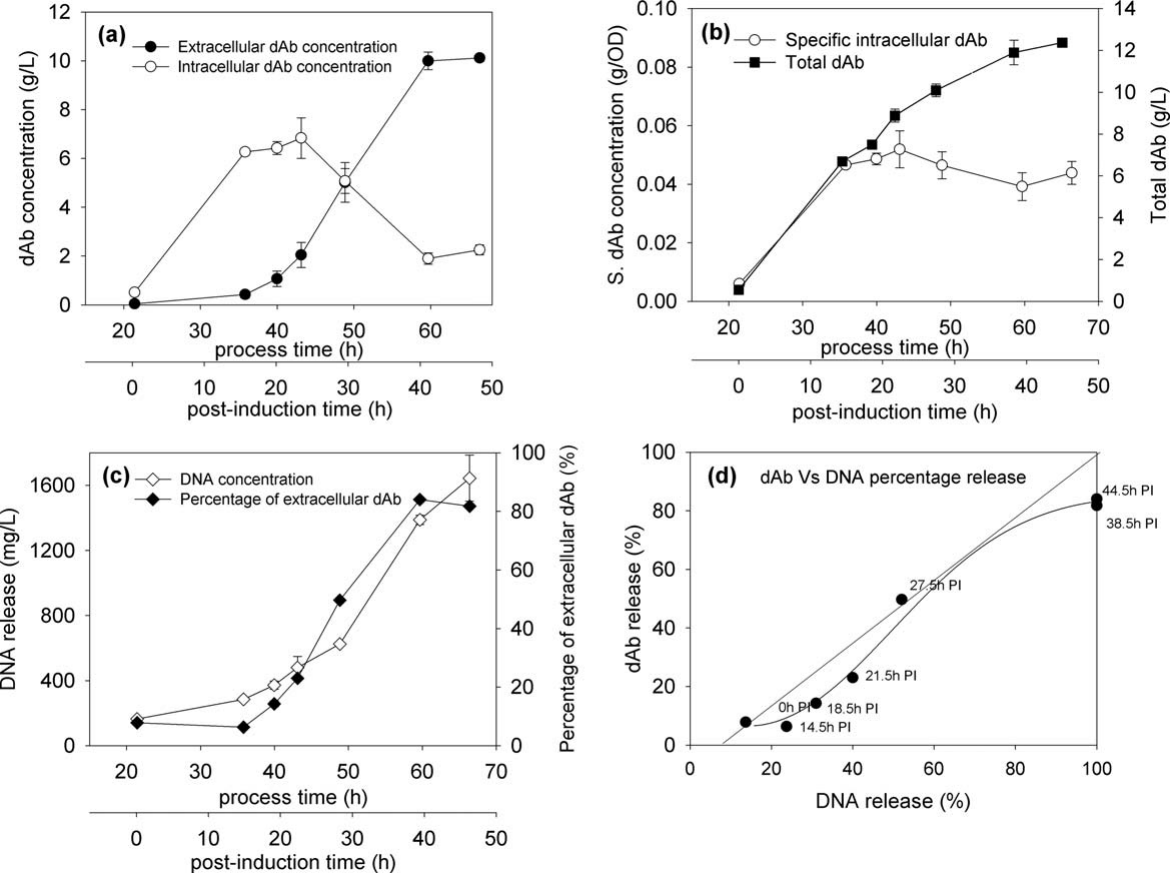
**Representative dAb and DNA profiles for 1 L fermentation studies.** Given are (a) extracellular and intracellular dAb concentration; (b) specific intracellular and total dAb concentration, (c) %dAb and DNA concentration in culture supernatant; (d) parity plot comparing release into extracellular environment of both dAb product and DNA. The parity line allows for the initial presence of DNA at time of induction (equivalent to 12% release) and the line then shows relationship if dAb and remaining DNA are released to equal extents. To note, 100% dAb release refers to maximum product available at that point in fermentation.

The output of a fermentation process for the dAb preparation is given in terms of cell biomass and metabolism (Figure 2) and dAb formation and location and DNA release (Figure 3). All descriptions of the fermentation process will be in terms of time post induction. Peaks are observed at ∼15 h for CER and OUR (Figure 2a) with the resultant RQ values ranging initially from 0.90 to finally 0.95 signifying growth primarily on glycerol rather than yeast extract proteins. Similarly peaks are observed at 15 h for OD_600_ and dcw (Figure 2b) and capacitance (Figure 2c). This is indicative of the onset of cell lysis and loss of cell biomass. Following the onset of cell lysis there is a significant increase in the broth viscosity (Figure 2c) which is reflected in a decrease in the ratio of measured *K*_L_ *a* (*K*_L_ *a*_m_) as derived from the recorded OUR values^35^ to predicted *K*_L_ *a* (*K*_L_ *a*_p_) as measured using media before inoculation from changes in DO as a function of changes in stirrer speed and gas flowrate. An increase in broth viscosity will lead to an underestimate of *K*_L_ *a*_p_ and hence a reduction in the ratio of *K*_L_ *a*_m_/*K*_L_ *a*_p_ as observed in Figure 2a from 20 h onwards. It appears that cell lysis might be complete after ∼40 h with no further decrease in OD_600_ (Figure 2b) or in capacitance (Figure 2c). Increases observed in wcw or dcw (Figure 2b) are probably indicative of the challenges of recovery and washing of cell pastes once high levels of lysis have occurred, for example, leading to a highly viscous broth (Figure 2c) [the lack of response in wcw values to lysis (Figure 2b) is probably also for reasons of difficult dewatering during cell paste washing].

The relative amounts of extracellular and intracellular dAb (Figure 3a) follow the expected pattern for a broth where dAb is mainly released from the cells by lysis with the onset being as described for Figure 2. The total dAb formed (Figure 3b) is the accumulative effect of viable cells present. The constant level of specific intracellular dAb (Figure 3b) is probably a reflection of the limit of cell capacity for dAb to be held in the periplasmic space. As might be expected for a process where cell lysis is the dominant factor controlling release, the relative amounts of dAb and DNA appearing in the broth parallel each other closely both in terms of trends with time of fermentation (Figure 3c) and the resultant parity plot (Figure 3d).

Fermentations with a working volume of 20 L were carried out with the intention to mimic the 1 L scale fermentations and provide material for pilot‐scale studies. Scale up from the 1 L scale was on the basis of control to the same DO levels and operation to the same extent of cell lysis. Table 1 summarises the performance data for the 1 and 20 L scales. The fermentation biomass and productivity were reduced with scale up by ∼20–30%. This was attributed to greater fluctuations observed at larger scale of DO and not to inefficient mixing of the fermentation broth creating oxygen and substrate gradients^36^, ^37^ or to greater shear stress leading to cell damage.^36^ The greater peak biomass levels achieved at 1 L scale led to greater extents of DNA release and hence higher viscosity broths, and also higher dAb titres. A similar pattern of these inter‐relationships is observed comparing the results for the two 20 L scale fermentations. The same proportions of dAb release are observed for all fermentations studied.

**Table 1:** Comparison of Performance of 1 and 20 L Scale Fermentations

Performance metric at end of fermentation	1 L fermentations (*n* = 3)	20 L Fermentation 1	20 L Fermentation 2
Biomass‐OD_600_(peak value)	51.4 ± 1.4 (132)	67.7 (103)	52.5 (95)
DNA (mg L^−1^)	1644 ± 142	1290	1060
Broth rheology* *k*, × 10^3^ N s^*n*^ m^−2^; *n*)	129 (*n* = 0.66)	20.1 (*n* = 0.86)	4.91 (*n* = 0.95)
Total product (g L^−1^)	12.3 ± 0.3	7.8	7.2
Product release (%)	81.8 ± 0.5	82	83

*Power law fit of data.

**Figure 4:**
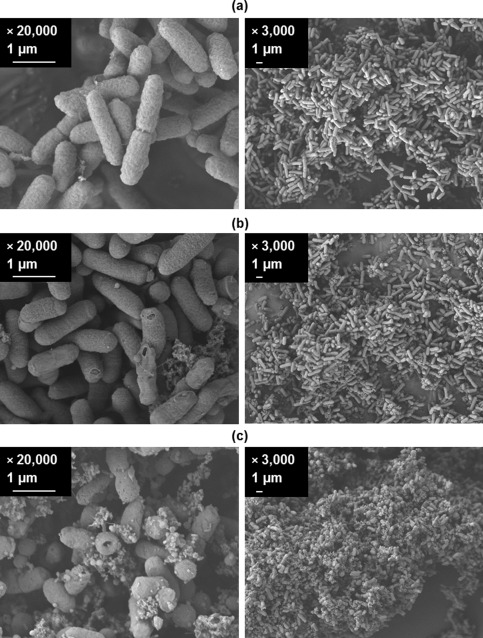
**Scanning electron microscopy images of fermentation samples.** 20 L scale, Fermentation 2 (Table 1) samples are for postinduction and (total process) times of: (a) 0 (19.3) h, (b) 17.7 (37.0) h, and (c) 42.6 (61.9) h.

A visual representation of what happens during cell lysis is given in Figure 4 using electron microscopy. The cells at point of induction appeared relatively unaffected by the fixing process to prepare the cells (Figure 4a). Close to the post induction time where cell lysis is presumed to start (Figure 2) the cells are now observed to be leaking intracellular content but without major disintegration of the overall cell membrane structure (Figure 4b). This trend continues till the end of fermentation where nearly all cells show evidence of having lysed (Figure 4c).

**Figure 5:**
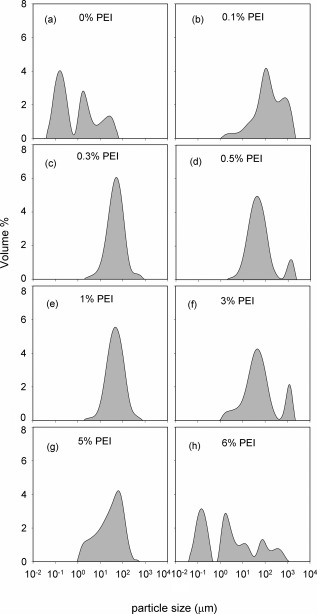
**Particle size distribution for PEI‐flocculated *E. coli* cell broth.** The ratio of cell broth to PEI solution is 24:1 for final PEI concentrations up to 1% w/v and 7.7:1 for higher concentrations. The PEI concentrations in the feed were varied from 0 to 50% w/v to give the final resultant overall PEI concentrations (w/v of broth as indicated in graphs). The material used for this study was autolysed broth collected at harvest (i.e., 49 h post induction). The concentrations of PEI g/g wcw used were (a) 0, (b) 0.006, (c) 0.018, (d) 0.030, (e) 0.065, (f) 0.195, (g) 0.325, and (h) 0.391.

The conditions to be used for flocculation of the cell broth were determined by use of particle size distributions to characterise the effect of concentration of PEI (Figure 5). The choice of PEI concentration is based on the removal by aggregation of particles in the submicron range i.e. those particles, which will be difficult to remove by centrifugation. A wide range of PEI concentrations achieves this; in particular the size distributions obtained with 0.1, 0.3, and 1% (w/v of broth) PEI, corresponding to 0.018, 0.030, and 0.065 g/g wcw, respectively, show essentially the same final low levels of particles below ∼2 micron. Overdosing to 6% (w/v of broth) PEI leads to restabilisation of the cell debris and hence of the submicron material.^38^ A concentration of 0.5% (w/v of broth) PEI is selected as a mid‐range value to cope with variations in fermentation broth, for example, due to changes in time of harvest or scale of fermentation. The broths studied here are such that the PEI concentration remains within the range of 0.018–0.065 g/g wcw where performance is optimum.

### USD primary recovery investigation

The USD studies were used to characterise the nature of the process material to be processed in terms of the ease of sedimentation of the solids in the suspension i.e. a compound property of density difference between the particles and the suspension, (size)^2^, (suspension viscosity)^−1^, solids volume fraction and shape factor etc. Some interpretations are given of these individual properties but many are challenging to characterise for biological materials.

**Figure 6:**
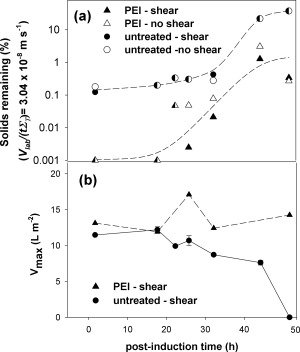
**USD shear stress, centrifugation and supernatant filtration studies.** (a) % solids remaining (Eq. 2) after USD centrifugation for untreated and flocculated cell broth at 0 and at 0.53 × 10^6^ W/kg shear stress. Centrifugation conditions used: 6000 rpm for 7 min, max RCF 3,400*g*, *Σ*_T_ = 0.16 m^2^ (see Eq. 1), *V*_lab_ */tΣ*_T_ = 3.04 × 10^−8^ m/s (0.011 L/m^2^/h). Lines are best fit by eye. (b) Depth filtration studies of supernatant from untreated sheared broth (*n* = 2) with *V*_max_ evaluated using Eq. 3; supernatant from flocculated sheared broth (low values only – see text), *n* = 1. The volumetric concentration of the PEI used was 0.5% (w/v of broth) giving PEI concentrations ranging from 0.028 to 0.036 g/g wcw.

The 1 L scale fermentation (Figures 2 and 3) was used as the basis for USD studies to predict the effect of harvest time and of flocculation on the ability to clarify the broth by full‐scale continuous‐flow disc‐stack centrifugation followed by depth filtration (Figure 6). For untreated broth good clarification by centrifugation was achieved until significant cell lysis had occurred such that the changes to the suspension properties of viscosity (Figure 2d) and the release of intracellular components (Figure 2d) led to poor clarification (Figure 6a). The effect of shear stress as might occur in the feed zone of a high shear stress non‐hermetic centrifuge is negligible in terms of loss of clarification. The filtration capacity of the clarified broth (Figure 6b) is significantly diminished as the solids carry‐over increases. The filter capacity is based on the assumption that the filter operation is determined by the pore constriction model as evidenced by observation of the loaded filter and by the use of various data plots, for example, *t*/*V* versus *t* or versus *V* plots (not shown here) to test for the applicability of the pore constriction or resistance models, respectively.^31^, ^32^ For PEI‐flocculated broth, there is a major increase in solids removal and again this performance is independent of the application of high shear stress. The centrifugal clarification for PEI‐flocculated broth was improved in all instances compared with untreated broth. The postinduction time after which recovery of untreated broth becomes significantly more difficult (27.5 h) corresponds to the point in Figure 3c where the extent of DNA release, and hence viscosity, increases sharply. This translates to significant decreases in the projected filter capacity required to process the supernatant. PEI precipitation/flocculation leads to supernatants which do not follow the pore constriction or resistance model as evidenced from the highly nonlinear plots of *t*/*V* versus *t* or *V* and where a high variation of performance was noted (+/−30%). Insufficient samples were available for a large number of repeats; the results reported are for the minimum values of capacity estimated by observation of the final data points where flux had essentially ceased, that is, the value which might be used to ensure robust operation. In contrast to the trend observed for untreated broth, no reduction in filter capacity is noted with increased postinduction times.

**Figure 7:**
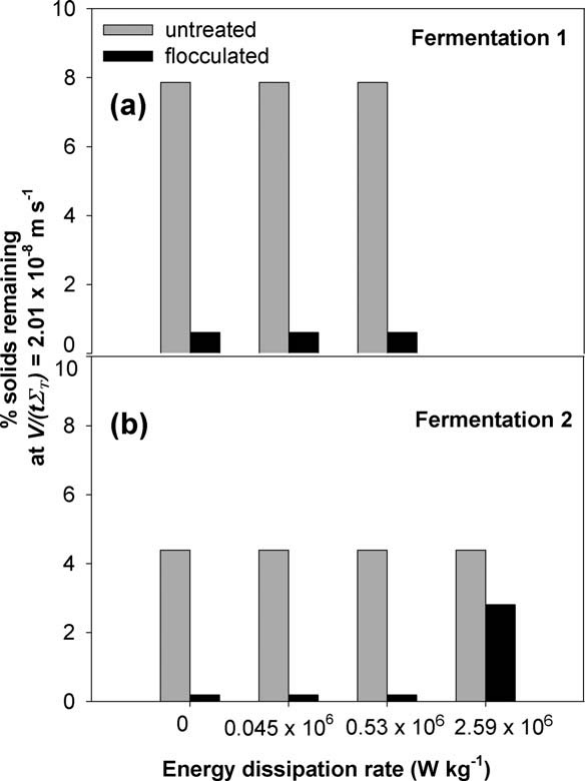
**USD shear stress and centrifugation studies for clarification of untreated and flocculated broths produced at 20 L fermentation scale–effect of energy dissipation rate.** Centrifugation conditions used 8000 rpm for 6 min, max RCF 6,010*g*, *Σ*_T_ = 0.28 m^2^ (see Eq. 1). *V*_lab_ */tΣ*_T_ = 2.01 × 10^−8^ m/s (equivalent to 0.072 L/m^2^/h). The PEI concentration of the flocculated broth was 0.5% (w/v of broth), giving PEI concentration of 0.033 and 0.041 g/g wcw for Fermentation 1 and 2, respectively.

The centrifugation conditions used in Figure 6 were as for an industrial centrifuge operating at midrange flowrate of its capacity. This leads to supernatants of untreated broth at the end of fermentation with unacceptably high solids content (∼21% solids carry over). The USD studies for the final broths prepared at 20 L scale used an increased (by ∼50%) extent of centrifugation, that is, as for industrial centrifuge operating at the low flowrate range of its capacity (Figure 7). Such a change is predicted to lead to a halving of solids carry over.^22^ The overall effect combined with the reduced final viscosities (Table 1) led to improvements in clarification of untreated broth from 21 to 4–8% solids carry over with the higher viscosity broth, that is, with greater DNA release, leading to the higher level of solids carry over. As for the previous studies the solids carry over is not affected by the application of even very high shear stress levels. The effect of flocculation is again to reduce significantly the percentage solids carry over, which again is independent of exposure to shear stress except at the very highest levels (2.59 × 10^6^ W/kg) imparted. The USD predictions for the recovery of flocculated broth from Fermentation 2 were carried forward for comparison with pilot‐scale recovery.

### Pilot‐scale verification

The conditions used for the pilot‐scale studies of centrifugation followed by filtration were based on the USD studies (Figures 6 and 7) where the indication is that low throughput conditions should be used to process the inherently challenging process material with *Q*/*Σ* value of ∼2 × 10^−8^ m/s and filter flux rate of 100 L/m^2^/h.

**Figure 8:**
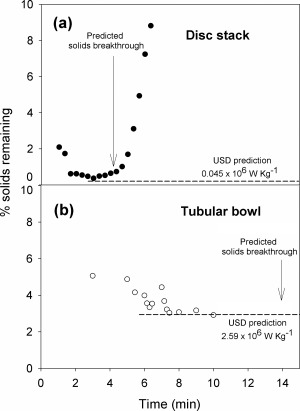
**Pilot‐scale centrifugation of flocculated fermentation broth.** Centrifuges used and operating conditions:(a) Disc stack centrifuge of solids holding capacity 0.25 L, *Σ*_(__*c*__= 0.4)_ *=* 680 m^2^, operated with a low shear stress hydro‐hermetic feed zone at 50 L/h to give (*Q/Σ*)_ds_ = 2.01 × 10^−8^ m/s (0.072 L/m^2^/h) and time to steady state predicted at 3.6 min (b) a tubular bowl centrifuge (CARR Powerfuge) of solids holding capacity 1.0 L, *Σ*_(__*c*__= 0.9)_ *=* 930 m^2^) operated at 60 L/h to give *(Q/Σ*)_tb_ = 1.78 × 10^−8^ m/s (0.064 L/m^2^/h), and time to steady state predicted at 5.5 min. The dashed lines indicate the USD prediction (from Figure 8) for given energy dissipation rates. Supernatant for filtration studies (Figure 9) is collected between predicted time to steady state and the predicted time to solids breakthrough.

The performance of a disc stack centrifuge and a tubular bowl centrifuge for the clarification of the flocculated material characterised in Figure 7b is presented in Figure 8. The disc stack operates with a low shear stress hydro‐hermetic feed zone which has been shown^22^ to equate to 0.045 × 10^6^ W/kg being imparted to the process material. For equivalent values of (*Q/Σ*)_ds_ for the disc stack to the *V*_lab_ */(tΣ_T_)* value used in USD studies there is good agreement between pilot‐scale performance and USD prediction for the % solids recovery. Similarly the breakthrough prediction based on a volume balance and the sediment hold up space gives a good estimate of the actual breakthrough.

For the tubular bowl centrifuge, the feed zone for the machine design used here is assumed to expose feed material to a very high shear stress.^28^ The pilot‐scale centrifuge had to be run at a *(Q/Σ)*_tb_ value slightly lower than the *V*_lab_ */(tΣ_T_)* value used in the USD study; however, it is predicted from previous work^22^ that there will be only a small (∼10%) increase in % solids carry over for the higher USD *V*_lab_ */(tΣ*_T_ *)* value used here. There is good agreement between the USD prediction and the performance of the pilot‐scale solid bowl centrifuge. Insufficient material was available to allow a test of the prediction for breakthrough.

**Figure 9:**
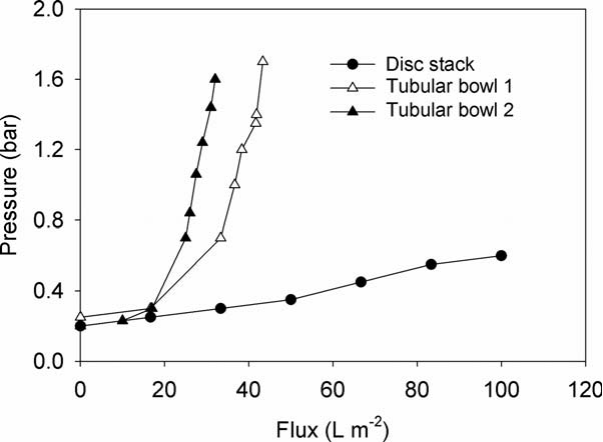
**Filtration of supernatant from pilot‐scale centrifugation of flocculated broth.** See Figure 8 for details of supernatant preparation using: (▴,▵) solid bowl tubular centrifuge (Powerfuge); (•) disc stack centrifuge. Filtration was by 0.1 m^2^ depth filter, pore size 0.05–0.2 μm, operated at 100 L/m^2^/h.

The filtration of supernatants produced is compared in Figure 9. A *V*_max_ of ∼15 L/m^2^ for the supernatant produced using the tubular bowl centrifuge is similar to the USD prediction for *V*_max_ (Figure 6) for centrifuged flocculated broth produced at the end of fermentation with both feeds having similar solids content. For the supernatant prepared using the disc‐stack centrifuge, the 6‐fold greater clarification achieved compared to the tubular bowl centrifuge might be expected to lead to the significantly higher (at least 6‐fold) *V*_max_ value observed in the filtration study (Figure 9).

## Discussion

**Figure 10:**
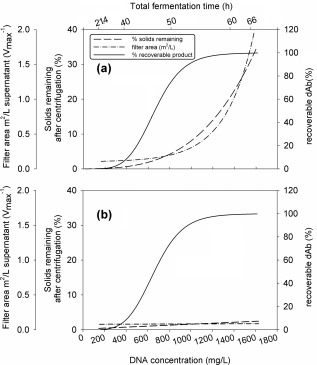
**Relationship between dAb and DNA release, solids remaining after centrifugation and depth filter area for (a) untreated and (b) flocculated broths.** Here 100% dAb recovery refers to total extracellular dAb available after fermentation time of 66 h. Centrifugation conditions for equivalent *Q/Σ* = 3.04 × 10^−8^ m/s (0.011 L/m^2^/h) for a high shear stress, nonhermetic feed zone.

The challenge of processing an *E. coli* broth may be approximated to the extent of DNA release and this is summarized in Figure 10. Here the extent of product released to the broth, and hence yield of the process, increases with the extent of DNA release. The latter largely determines the broth viscosity and hence the performance of a centrifuge for broth clarification. The extent of solids carry over in a pilot scale centrifuge is shown as a prediction from the USD tests (Figure 6). This solids carry over in turn affects the performance of a depth filter for further clarification and again USD predictions of filter area required are given. For untreated broth, at DNA release levels >∼600 mg/L, the solids carry over and hence filter area required starts to rise considerably. Processing options might include the installation of greater centrifugation and filtration capacity or the sacrifice of product yield by stopping fermentation early. For this second option the challenge is to be able to monitor the fermentation to predict the best time to harvest. Some monitoring options, which might be used are explored in Figure 2 and include the profiles of capacitance, viscosity, OD_600_, and dcw.

The use of flocculation both to precipitate the DNA and to flocculate the resultant solids (precipitate, debris, etc) results in the removal of submicron particles. From Figures 5 and 6, this appears to be a relatively robust process with good removal of fine particles over a range of concentration ratios of PEI to wcw. The USD prediction for the processing of flocculated end‐of‐fermentation broth is of good solids removal by centrifugation resulting in a filterable supernatant and ∼100% yield of the dAb. The key process variable requiring control is the extent of shear stress to which the flocculated broth is exposed, for example, in the feed zone to a continuous‐flow centrifuge. The prediction is that this is only of concern at extremely high shear stress levels (Figure 7). This is verified at pilot scale for the high‐speed continuous‐flow tubular bowl centrifuge (Figure 8) followed by supernatant filtration (Figure 9). It is noted that tubular bowl centrifuges equipped with low energy dissipation rate feed zones are available to process such materials as studied here with reduced levels of break up. The verification at pilot scale does depend on the ability to scale up the flocculation preparation stage prior to recovery by centrifugation and filtration. In general this might be achieved by maintaining the same mean power dissipation per unit volume (or mean velocity gradient) and time of ageing (e.g., as in Boychyn et al.^28^). The effect of PEI flocculation/precipitation on the presence of key contaminants has been characterised for the same system as studied here^2^ with over 4‐fold reduction of DNA, 1.5‐fold reduction of host cell proteins, no increase in endotoxins levels and no loss of dAb. Even better purification enhancement might be achieved by adjustment of the harvest point, this is for future consideration.

## Conclusion

This study demonstrates that with limited ∼100 mL samples of fermentation broth valuable insight may be gained into a sequence of full‐scale processing options including flocculation, centrifugation and depth filtration. In this way a USD platform can be used to help select the fermentation point of harvest most suitable for recovery processes and also help identify early, options suitable to take forward to full scale studies. There is a range of process variables which might be further studied using USD methods e.g. the hold conditions and times of the broth after fermentation or after flocculation or the time taken and conditions used for flocculation. The operating parameters studied here are as might be used in a pilot plant with little to no delay time in processing. Evidently for process robustness, significantly longer times might be usefully studied e.g. to explore if further cell lysis or product degradation etc. might occur.

The USD studies also may provide a base to examine the effect of processing and especially of reagent (PEI) addition on the quality attributes of the product. Brief studies previously reported^22^ showed no impact on the aggregation of the dAb but these would evidently need to be extended. There would also be a need to demonstrate control of PEI reagent remaining after centrifugation and filtration. In this study PEI addition was determined as a fixed level per unit volume of broth. Changes in cell density and extents of lysis might require adjustment of the levels used. This is difficult to predict without experimental studies especially as the mechanism of action is a complex combination of flocculation and precipitation. Recent advances in PEI analysis might provide scope for the monitoring of residual PEI.^39^
